# Exploring phase contrast imaging with a laser-based K_α_ x-ray source up to relativistic laser intensity

**DOI:** 10.1038/s41598-020-63614-3

**Published:** 2020-04-21

**Authors:** M. Gambari, R. Clady, A. Stolidi, O. Utéza, M. Sentis, A. Ferré

**Affiliations:** 1 Aix Marseille Université, CNRS, LP3, 13288 Marseille, France; 2 CEA, LIST, Department of Imaging and Simulation for Non-Destructive Testing, F-91191 Gif-sur-Yvette, France

**Keywords:** Imaging techniques, X-rays, Laser-produced plasmas

## Abstract

This study explores the ability of a hard K_α_ x-ray source (17.48 keV) produced by a 10 TW class laser system operated at high temporal contrast ratio and high repetition rate for phase contrast imaging. For demonstration, a parametric study based on a known object (PET films) shows clear evidence of feasibility of phase contrast imaging over a large range of laser intensity on target (from ~10^17^ W/cm^2^ to 7.0 × 10^18^ W/cm^2^). To highlight this result, a comparison of raw phase contrast and retrieved phase images of a biological object (a wasp) is done at different laser intensities below the relativistic intensity regime and up to 1.3 × 10^19^ W/cm^2^. This brings out attractive imaging strategies by selecting suitable laser intensity for optimizing either high spatial resolution and high quality of image or short acquisition time.

## Introduction

The interest of developing new x-ray sources and/or improving their performances in terms of brightness, stability, and compactness is still growing since decades. This is strongly motivated by applications of x-ray sources for imaging and related applied developments to biology, medicine and material science. In particular, the advent of synchrotron radiation sources in the seventies as well as the development of optical components for x-rays, definitively allowed to transfer the phase contrast imaging (PCI) techniques from the visible spectral range to the x-ray one. Phase contrast x-ray imaging is sensitive to phase shift induced by an object placed in the x-ray path and does not rely on its absorption. Thus, it can image weakly absorbing materials, such as carbon-based materials and biological objects. In addition, it should be noted that the sensitivity of absorption contrast decreases as the photon energy (E) increases^1^ as E^–3^, whereas that of phase contrast methods decreases only as E^–2^. Therefore, phase contrast methods are more sensitive at high photon energies (E = 10 to 100 keV), compared to absorption methods. In that case, for comparable image quality, the absorbed x-ray dose is smaller than with conventional radiography. Challenges addressed by hard x-ray PCI are numerous such as the detection of complex damages in composite materials^2–4^ or of the apparition of microcalcifications around a hundred micrometers of diameter at an early stage of breast cancer^5–7^.

X-ray sources with high spatial coherence and photon flux required for PCI^8^ are a difficult technological realization. On one hand, conventional microfocus^9–11^ and liquid-metal-jet^12^ x-ray tubes are very compact and inexpensive sources. Even if liquid-metal-jet x-ray tubes tend to increase their photon flux, both sources are continuous excluding time-resolved studies. On the other hand, synchrotrons^13–16^ offer today the highest brightness available and are suitable for imaging applications. However, they are very large infrastructures with limited access, high cost and currently not scalable to civil environment such as hospitals. Between these two alternatives, ultrafast x-ray laser plasma sources appear as good candidates for PCI at a laboratory scale. Among them, x-ray sources provided by laser plasma acceleration such as Betatron^17–23^ and inverse Compton scattering^24–27^ offer potential alternatives thanks to their high brightness and very small source size, making possible the acquisition of an x-ray image in single shot mode. However, they are until now based on laser driven systems with high peak power (>30 TW)^23^ difficult to scale up to high repetition rate. Moreover, these sources provide collimated x-ray beams at low divergence limiting the field of view. In this context, an attractive solution is the K_α_ x-ray table-top source driven by femtosecond laser systems^28,29^.

Hard K_α_ x-ray source is generated by interaction between an intense femtosecond laser pulse (I ≥ 10^16^ W/cm^2^) with a high Z solid target. The spectrum is composed of a large x-ray Bremsstrahlung emission, dominated by K_α_ line (up to ~50%)^30,31^, characteristic of the solid target material. In addition, it is spectrally tunable by changing the target material. This can give access to high energetic K_α_ photons (>20 keV) for high Z material like silver, tantalum or tungsten, difficult to reach by other kind of x-ray sources while combining a high flux. Finally, this source also has a pulse duration below one picosecond^32^, suitable to perform time-resolved applications. With the new advanced driving laser sources, brightness comparable to the 3^rd^ generation of synchrotrons has been demonstrated^29,30^. Previous studies have shown the capacity of this source for PCI using the method of in-line or propagation-based phase contrast imaging (PBI)^31,33,34^ for biological samples^30,35–39^, for shock wave characterization in the context of fusion plasma^40^, or for material characterization at submicron scale^41^. These demonstrations have been mainly performed with low or kHz repetition rate laser systems for a fixed laser intensity without investigating the impact of this parameter on the PCI feasibility. Therefore, in this manuscript, a parametric PCI study with the PBI method is performed to show the capabilities of K_α_ x-ray laser plasma source on a large range of laser intensity. Experimental demonstration is done for a reference low absorbing sample made of PET (polyethylene terephthalate, a carbon-based material) films of different thicknesses and for a wasp as an example of a biological sample. For the first time, PCI study including retrieved phase images is realized at a very high laser intensity regime on target (up to ~10^19^ W/cm^2^) with 100 Hz laser repetition rate. The study highlights that it is possible to obtain a retrieved phase image with a noticeable reduction of acquisition time even at very high driving laser intensity. The counterpart is a degradation of image quality because of the enlargement of the x-ray source size. Such advance paves the way for future applications where the acquisition time is a crucial parameter.

### A unique laser system for exploring phase contrast imaging

**Figure 1:**
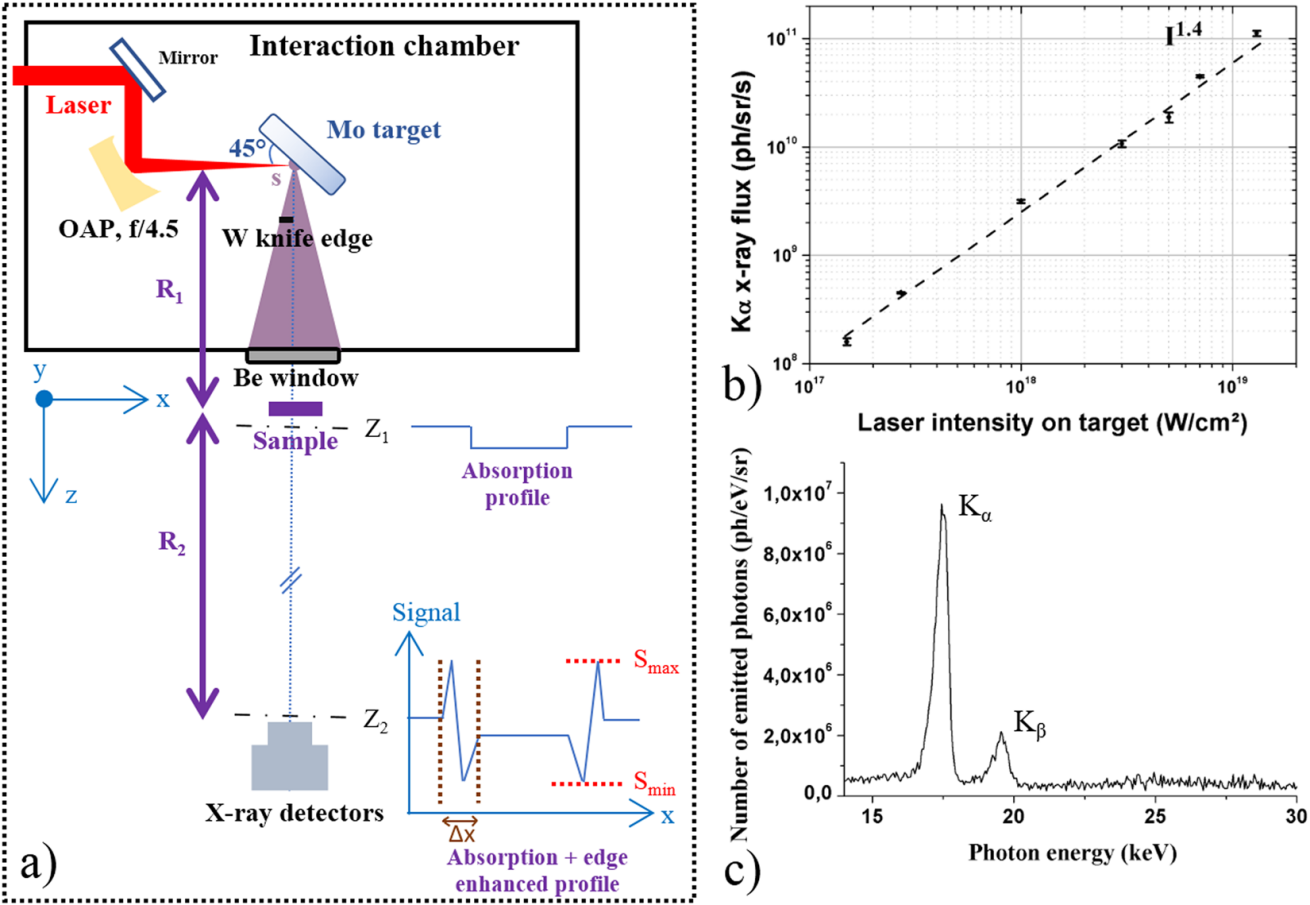
(**a**) Experimental setup. A knife edge made of tungsten is positioned between the Mo target and the detector, at R_knife_ = 6 cm from the x-ray source, to measure the effective x-ray source size (see *Methods*). The interaction chamber is under a residual pressure of ~10^−4^ mbar and equipped with a beryllium window of 800 µm thickness. The imaged sample is located at R_1_ = 32 cm from the x-ray source. The x-ray detector is placed at R_2_ = 60 cm or 90 cm from the sample. (**b**) K_α_ x-ray flux versus laser intensity on target. Dots correspond to the experimental points which are an average of at least three measurements of the x-ray flux. The error bars are the standard deviation of these measurements. A power law dependence (dot line) between laser intensity and K_α_ x-ray flux, ${{\rm{N}}}_{{{\rm{K}}}_{{\rm{\alpha }}}}\propto$ I^ε^ is also shown, with ε = 1.4 in accordance with our previous study^29^. (**c**) Example of Mo K_α_ x-ray spectrum.

To evaluate the interest of K_**α**_ x-ray sources generated with ultrahigh laser intensity for fast PCI applications, we perform the experiments with the 100 Hz, 10-TW, 800 nm laser beamline^42^ of ASUR facility, a unique laser combining high average and high peak power. The p-polarized laser beam of ~25 fs pulse duration is focused on a molybdenum (Mo, ${{\rm{E}}}_{{{\rm{K}}}_{{\rm{\alpha }}}}$ = 17.48 keV) thick target at an angle of incidence of 45° with an off-axis parabola (Fig. 1a). More details on the target surface can be found elsewhere^29^. The focal spot diameter is ~6 µm (FWHM) with the encircled energy at FWHM measured at ~34%, and the maximum energy of ~130 mJ is delivered on target. Considering the real 2D beam profile, it corresponds to a maximum peak intensity of 1.3 × 10^19^ W/cm^2^. The present study is done with a high temporal intensity contrast ratio (ICR) laser pulse^29^ ~10^10^ which is beneficial to produce a high x-ray flux^43^ and at the same time a small x-ray source size^44^. Since x-ray flux and source size are critical parameters to enhance PCI, they are thoroughly characterized for all of these studies and investigated by varying the laser intensity in a large range (from ~10^17^ to 1.3 × 10^19^ W/cm^2^, see Fig. 1b). An example of a measured x-ray spectrum, thanks to a direct-detection back illuminated CCD camera, is shown in Fig. 1c.

For PCI evaluation, an experimental setup (Fig. 1a) is developed, based on the PBI method. This method does not require optics^45^, ensuring simplicity and preserving enough photon flux on the detector. A sample with a refractive index n = 1 − δ + iβ with δ and β the real and imaginary parts respectively, is introduced in the x-ray path at the distances R_1_ from the source and R_2_ from the detector. The phase shift ϕ introduced by the x-ray wave travelling through the object compared to a non-scattered wave results from the variation of δ. Quantitatively, the recorded intensity profile S(x, y, z) by the detector is described by the simplified transport of intensity equation for a weak absorbing object and unit-amplitude plane wave illumination^46^ S(x, y, z) $\approx \,1+\frac{{\rm{\lambda }}{\rm{z}}}{2\pi }{\nabla }_{\perp }^{2}{\rm{\phi }}(x,y,0)$ with λ the wavelength and z the propagation distance. This method enhances visualization of sample contours, optical index transition or abrupt thickness variation of low absorbing objects thanks to the detected phase curvature ${\nabla }_{\perp }^{2}\phi$. To evaluate the phase information contained in this equation, the parameter C_edge_ is defined as^28,30^ $\frac{{{\rm{S}}}_{{\rm{\max }}}-{{\rm{S}}}_{{\rm{\min }}}}{{{\rm{S}}}_{{\rm{\max }}}+{{\rm{S}}}_{{\rm{\min }}}}\times 100 \%$ where S_min_ and S_max_ correspond to the minimum and maximum intensities of the detected edge enhanced contrast (Fig. 1a). To maximize C_edge_ (%) a high spatial coherence length^10^ ${{\rm{L}}}_{\perp }=\frac{{{\rm{\lambda }}{\rm{R}}}_{1}}{s}$ of the x-ray source is required. Thus, a small x-ray source size s is necessary. Otherwise, only an absorption image, related to β, can be acquired with enough accuracy (Fig. 1a). In addition, the diffraction of the x-rays induced by the object has to be considered from the object exit to the detector. Indeed, enough propagation distance z from the sample to the detector is desirable in order to be sensitive to the phase changes. For this reason, a parameter called shearing length^47^ L_s_ = $\frac{{{\rm{\lambda }}{\rm{R}}}_{2}|{\rm{u}}|}{{\rm{M}}}$ is introduced, where M = $\frac{{{\rm{R}}}_{1}+{{\rm{R}}}_{2}}{{{\rm{R}}}_{1}}$ is the magnification and ${\rm{u}}$ the spatial frequency component of the object. The latter is determined from the experimental estimation of the edge contrast signal spreading Δx (see Fig. 1a); u = $\frac{1}{2\Delta {\rm{x}}}$ is expressed in line pair per millimeter. This parameter takes into account the Fresnel diffraction until the measurement plane, so the contribution of the sample itself and the resolution of the detector. If the ratio L_s_/${{\rm{L}}}_{\perp }$ ≪ 1, the wavefront is almost fully coherent over the shearing length, and the phase curvature associated with the structure component is visible. Otherwise, if L_s_/${{\rm{L}}}_{\perp }$≫ 1, the wavefront is incoherent over the shearing length, and the phase curvature associated with the structure component is invisible.

## Results and Discussion

### Impact of laser intensity on C_edge_ values

**Figure 2:**
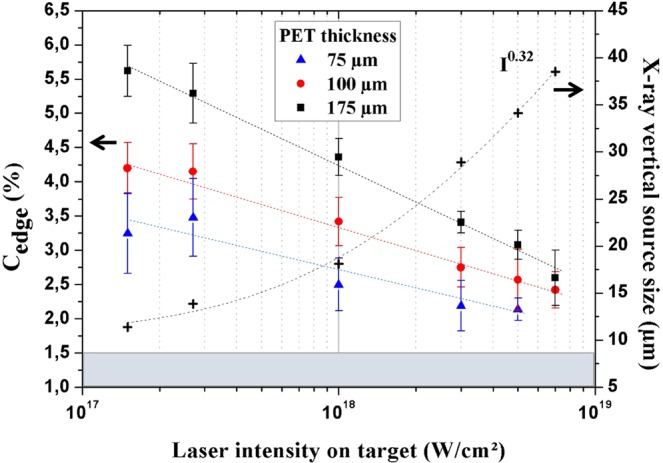
Evolution of C_edge_ and effective x-ray vertical source size as a function of laser intensity on target, for three different thicknesses of PET films (75 µm, 100 µm and 175 µm). Due to the vertical transition between PET sheets and air, only the vertical x-ray source size is relevant in the observed signal. Dashed line for x-ray source size data is the I^γ^ power fit with γ = 0.32. C_edge_ is an average of 5 values issued from different zones of the image (see *Methods*, Fig. 7a). Error bars for C_edge_ are the standard deviation of these values. The noise level C_noise_ on the camera related to the detection of the x-ray signal has been measured at ~1.5% (shadowed area). Dashed lines are guides for the eyes.

To highlight the feasibility of laser-produced plasma K_α_ x-ray sources for PCI in a wide range of x-ray flux and source size, a parametric study is performed on PET (C_10_H_8_O_4_) films by measuring C_edge_ as a function of laser intensity. PET real and imaginary parts of the refractive index are respectively^48^ δ = 9.8 × 10^−7^ and β = 5.3 × 10^−10^ at ${{\rm{E}}}_{{{\rm{K}}}_{{\rm{\alpha }}}}$ = 17.48 keV. The ratio δ/β, equal to ~1.8 × 10^3^, is highly favorable for phase contrast imaging. The sample is made of different PET thicknesses (75 µm, 100 µm and 175 µm) to evaluate the applicability of the PBI method to image thin objects. The sample is placed at R_1_ = 32 cm from the x-ray source and the detector at R_2_ = 60 cm from the sample (Fig. 1a). The study of the evolution of C_edge_ and of the effective x-ray source size is presented in Fig. 2 as a function of laser intensity from 1.5 × 10^17^ to 7.0 × 10^18^ W/cm^2^. The increase of laser intensity on target induces an enlargement of the x-ray source size (s $\propto$ I^γ^ with γ = 0.32) as discussed previously^28,29,49^. Thanks to the high temporal intensity contrast ratio of the laser pulse (~10^10^) and the good pointing stability (less than 1 µm displacement for a focused diameter of 6.2 µm at 1/e^2^) of the laser source associated with a precise targetry positioning system^29^, this increase is limited to a factor ~3.5 (11 µm $\le$ s $\le$ 38 µm). Since the number of produced x-ray photons varies with laser intensity (Fig. 1b), the number of shots for each image acquisition is adjusted (from 20 000 for the lowest laser intensity to 400 for the highest) in order to be able to detect C_edge_. With the limitation of the detector resolution, the extraction of the spatial frequency u gives similar values for the three thicknesses. Therefore, L_s_ is identical for all samples. The ratio L_s_/${{\rm{L}}}_{\perp }=\frac{({\rm{M}}-1){\rm{s}}|{\rm{u}}|}{{\rm{M}}}$ is then largely governed by the evolution of the transversal coherence length with laser intensity. With the measured Δx values (as described in Fig. 1a) estimated to 120 $\pm$ 20 µm and M = 2.9, we calculate L_s_/${{\rm{L}}}_{\perp }$ = 0.03 for the lowest laser intensity and L_s_/${{\rm{L}}}_{\perp }$ = 0.10 for the highest. Since this ratio is always <1, the phase curvature can be detected for the range of laser intensity presently studied. Indeed, C_edge_ is all the time measurable and reaches a maximum value of 5.6% at low laser intensity (I = 1.5 × 10^17^ W/cm^2^) for an effective source size of 11 µm, which is only × 1.8 the laser focal spot size. Nevertheless, the parameter C_edge_ decreases on the whole range of intensity until the maximum intensity of 7.0 × 10^18^ W/cm^2^ reached in this experiment. This is due to the increase of the x-ray source size (38 µm, × 6.3 the laser focal spot size, at I = 7.0 × 10^18^ W/cm^2^) implying deterioration of the spatial coherence. Nonetheless, this limited growth of the x-ray source size enables to keep a relatively high spatial coherence to extract C_edge_ information. However, only the values of C_edge_ ~2.6% for the thickness of 175 µm and C_edge_ ~2.4% for the thickness of 100 µm are retrieved for this laser intensity on target. In the case of the thinnest sample tested (75 µm), the C_edge_ parameter cannot be evaluated being below the limit of a detectable signal (C_noise_ ~1.5%, Fig. 2, see *Methods*). These experimental results nicely complement recent 1D simulation works^28^ in which the general degradation of C_edge_ was shown in correlation with the increase of the x-ray source size for a single object of variable size.

As a first preliminary conclusion, high laser intensity enables a high x-ray flux (so a short acquisition time) but it induces a decrease of the spatial coherence of the x-ray source and thus a decrease of C_edge_ values. However, for a large range of laser intensity, the L_s_/${{\rm{L}}}_{\perp }$ ratios always are below unity which is a good indicator for the phase curvature visibility.

### Towards biological phase contrast imaging applications

**Figure 3:**
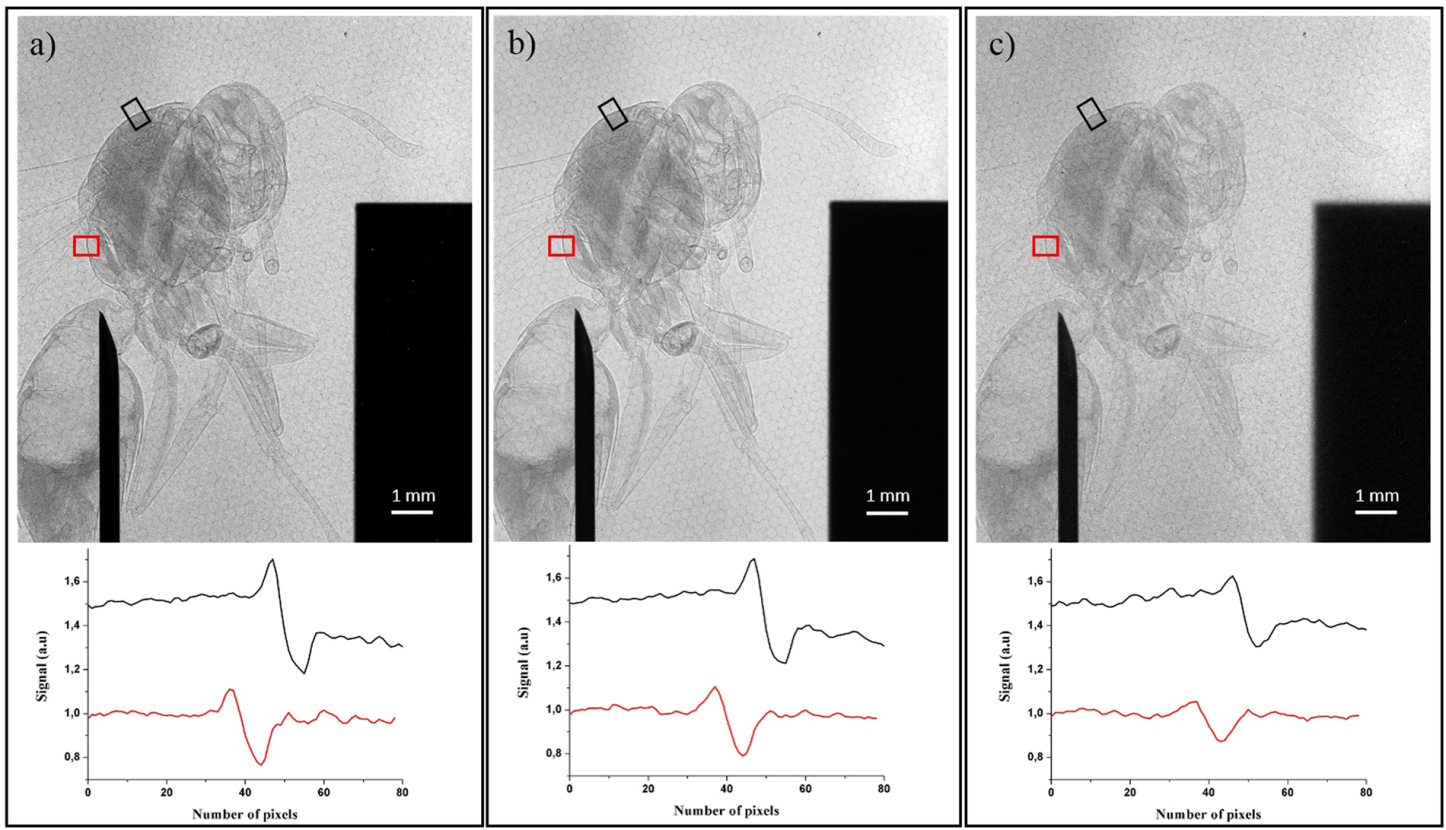
Raw x-ray phase contrast images of the wasp. The knife edge is simultaneously imaged to control the corresponding effective x-ray source size. Below each image are plotted two line profiles of the signal perpendicular to the edge for 2 thorax zones (black and red rectangles). (**a**) for I = 2.7 × 10^17^ W/cm^2^, (**b**) for I = 1.0 × 10^18^ W/cm^2^, (**c**) for I = 7.2 × 10^18^ W/cm^2^. The honeycomb structure in the background is related to the optical fibers which redirect the converted x-ray signal to the CCD array of the detector (see *Methods*).

Further on, to explore the capability of laser produced K_α_ source for applications, PCI of a wasp is performed for the first time for a large range of laser intensity and in the same experimental configuration. The distance R_2_ is increased to 90 cm in order to image the wasp on the entire detector surface and improve the phase contrast signal. The magnification is then M = 3.8. A comparison between raw phase contrast projection images of the wasp is shown in Fig. 3 for three different experimental conditions related to different laser intensities. It also includes for each image two line profiles of the signal perpendicular to the edge of two thorax zones, identical for the three images. The number of pulses for each image is varied (cf. Table 1) in order to obtain enough phase information to come out of the noise level. The choice of the laser intensity values (I = 2.7 × 10^17^ W/cm^2^, 1.0 × 10^18^ W/cm^2^, and 7.2 × 10^18^ W/cm^2^) corresponds to different trade-offs between access to short acquisition time (high intensity and moderate x-ray source size) and high image quality and information (low intensity and small x-ray source size).

**Table 1:** Laser intensities and corresponding x-ray source characteristics, spatial frequency u determined thanks to the edge contrast signal in Fig. 3 (red case) and the associated ratio L_s_/${{\rm{L}}}_{\perp }$. Spatial frequency u_body_ and corresponding ratio (L_s_/${{\rm{L}}}_{\perp }$)_body_ are given for a zone in the body of the wasp.

Laser intensity (W/cm^2^)	2.7 × 10^17^	1.0 × 10^18^	7.2 × 10^18^
Effective x-ray source size (µm)	11	18	38
K_α_ conversion efficiency (/2π sr)	2.85 × 10^−5^	6.76 × 10^−5^	1.04 × 10^−4^
Number of x-ray pulses	20000	8000	300
Spatial frequency u (lp/mm)	4	3	3
L_s_/${{\rm{L}}}_{\perp }$	0.032	0.045	0.084
Spatial frequency u_body_ (lp/mm)	7	5	4
(L_s_/${{\rm{L}}}_{\perp }$)_body_	0.056	0.066	0.11

Table 1 gives the effective x-ray source size and K_α_ conversion efficiency, and the number of x-ray pulses corresponding to the different laser intensities applied to obtain each image as well as the spatial frequency and the ratio L_s_/${{\rm{L}}}_{\perp }$.

**Figure 4:**
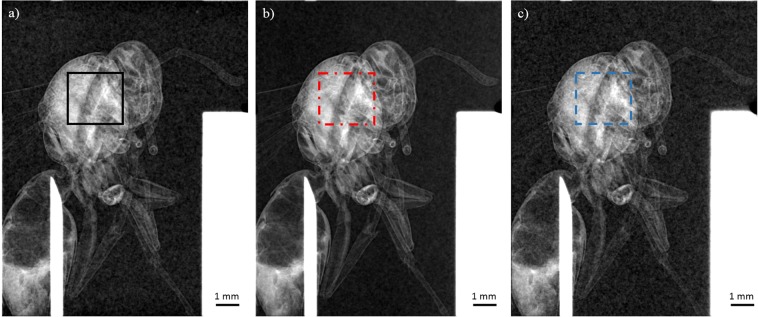
X-ray phase images based on Paganin phase retrieval algorithm^50,51^. (**a–c**) are retrieved from the raw images presented in Fig. 3 (**a–c**). (a) for I = 2.7 × 10^17^ W/cm^2^, (b) for I = 1.0 × 10^18^ W/cm^2^, (c) for I = 7.2 × 10^18^ W/cm^2^.

**Figure 5:**
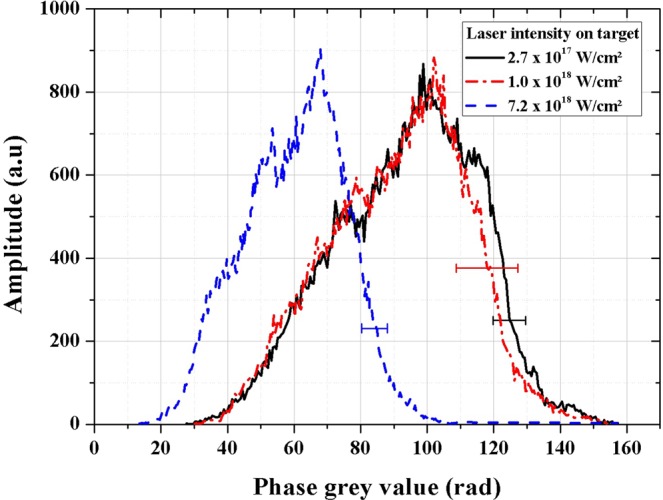
Phase grey histogram of region of interest from images (**a–c**) of Fig. 4. Error bars are correlated to the non-uniform response of the detector and take into account the (L_s_/${{\rm{L}}}_{\perp }$)_body_ ratios. They are similar for each point of the corresponding histogram.

For the wasp, the edge contrast is measured for all laser intensities. To support this, the L_s_/${{\rm{L}}}_{\perp }$ ratios are calculated for these three images on the edge contrast signal (red color in Fig. 3). They are constantly less than unity (Table 1). The edge contrast amplitude reduces with laser intensity due to the decrease of spatial coherence which is in excellent agreement with the results obtained on the PET reference sample. Nevertheless, we observe that the parameter C_edge_ is nearly the same between I = 2.7 × 10^17^ W/cm^2^ and I = 1.0 × 10^18^ W/cm^2^ (Fig. 3a,b) which is consistent with the limited increase of the x-ray source size in this intensity range (only by a factor 1.8). This is attractive in terms of decreasing notably the number of x-ray pulses required to obtain a raw image without altering significantly the edge contrast value. By increasing the laser intensity up to I = 7.2 × 10^18^ W/cm^2^ (Fig. 3c), the amplitude of the edge contrast signal is reduced almost by a factor of two. It is however still easily detected with only a limited number of shots to acquire a raw phase contrast image. These observations immediately translate into a reduction of the exposure time while obtaining an image of sufficient quality which can be a crucial issue for many applications. To go further, phase extraction based on Paganin approach^50^, and augmented by an implementation of the x-ray source size effect^51^, is applied on the wasp raw images (Fig. 3). For the wasp, the same composition than PMMA (C_5_H_8_O_2_) is taken, which is a good equivalent material for soft tissues^52^. Finally, an approximation of a monochromatic x-ray source is done. The retrieved phase images are presented in Fig. 4. The analysis is made on the center of the wasp where a region of interest (ROI) (in black solid line, red dashed dotted line and blue dashed line) is defined for each image. From these ROI’s, histograms of the retrieved phase grey values are plotted. The standard deviation of the histograms is a good parameter to evaluate the image dynamic. The latter is the ability of an imaging system to record a large range of the collected signal (here in Fig. 4 the phase signal). As for the raw images of the wasp, the quality of the retrieved phase extraction (red dashed dotted line and black solid line histograms in Fig. 5) is similar for I = 2.7 × 10^17^ W/cm^2^ (Fig. 4a) and I = 1.0 × 10^18^ W/cm^2^ (Fig. 4b). Indeed, the standard deviation σ for these two images are respectively σ_a_ = 21 rad and σ_b_ = 22 rad while σ_c_ =16 rad for I = 7.2 × 10^18^ W/cm^2^ (Fig. 4c). This dynamic degradation observed for the blue dashed line histogram in Fig. 5 can be explained by the decrease of the phase curvature contribution. Indeed, for this condition and for the body structure of the wasp, the (L_s_/${{\rm{L}}}_{\perp }$)_body_ ratio related to the body spatial frequency of the wasp u_body_ increases by a factor two compared to the case presented in Fig. 4a. This is due to the expansion of the source size (as commented previously) and because of the less phase curvature contribution related to a weaker definition of the body structure of the wasp (diminution of the u_body_ value, see Table 1). This also explains why the average phase grey value is weaker than in the cases presented in Fig. 5 (black solid and red dashed dotted lines). Therefore, the best trade-off laser intensity for phase extraction using Paganin algorithm is I = 1.0 × 10^18^ W/cm^2^ due to the reduction of the exposure time while keeping an excellent dynamic as discussed above.

**Figure 6:**
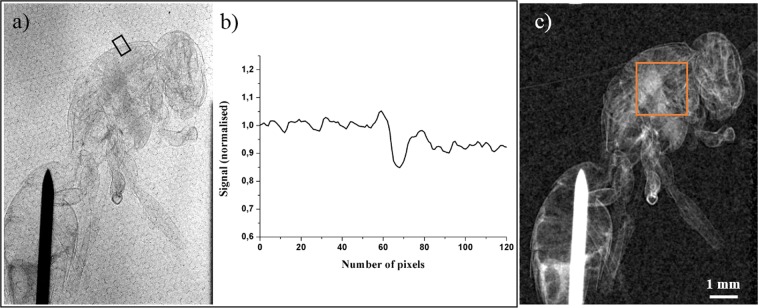
(**a**) Raw x-ray phase contrast image of the wasp acquired at I = 1.3 × 10^19^ W/cm^2^ and with 200 x-ray pulses. (**b**) Line profile of the signal perpendicular to the edge of a thorax zone. (**c**) Corresponding x-ray retrieved phase image.

To explore the limit of ASUR laser source, an ultimate x-ray PCI experiment has been carried out at the maximum laser intensity of I = 1.3 × 10^19^ W/cm^2^. The CCD camera is kept at 90 cm from the wasp and the raw image shown in Fig. 6a is obtained with an accumulation of only 200 x-ray pulses (2 seconds with 100 Hz driver laser). The effective source size of the x-ray source is 52 µm.

Compared to the previous images of Fig. 3 the quality of the raw image is undeniably degraded with less visible details. Nevertheless, the signal of the edge contrast still exceeds the noise level (Fig. 6b). This signal value is sufficient to extract a phase image shown in Fig. 6c for the air/wasp interface. For the body structure of the wasp, less phase curvature signal is also visible, involving a degraded phase extraction compared to previous images in Fig. 4, with an average grey value of 30 rad and σ = 15 rad (orange ROI in Fig. 6c). Even so, these last results demonstrate good applicability of our x-ray source and 100 Hz TW-class laser driver for phase-contrast imaging at high intensity and short exposure time.

## Conclusion

In summary, this study demonstrates the excellent ability for phase contrast imaging of a K_α_ x-ray source produced by a high repetition rate multi-TW class Ti:Sa laser system up to a very high range of laser intensity never experimentally explored. By a rigorous characterization of the x-ray source in terms of flux and effective source size done simultaneously for each PCI acquisitions, we clearly obtain raw phase contrast images and phase extraction with detectable phase information over the entire range of laser driver intensity explored from ~10^17^ W/cm^2^ to the maximum intensity tested of 1.3 × 10^19^ W/cm^2^. Thanks to the high temporal contrast of the laser system, the increase of the effective x-ray source size is restrained from 11 µm at 2.7 × 10^17^ W/cm^2^ to 52 µm at 1.3 × 10^19^ W/cm^2^. For PET samples as well as for the wasp a decrease of the edge contrast signal is observed when raising the laser intensity in agreement with a lowering of the x-ray source spatial coherence. The quality of the raw phase contrast image of the wasp is almost unchanged between 2.7 × 10^17^ W/cm^2^ to 1.0 × 10^18^ W/cm^2^ and with only ~10% loss of the C_edge_ factor and a stable dynamic and phase average value. Meanwhile, the number of laser pulses is reduced by a factor 2.5. At higher laser intensity, up to 1.3 × 10^19^ W/cm^2^, where a more pronounced increase of the x-ray source size happens, a degradation of the image quality is observed in comparison with PCI images obtained in a lower laser intensity regime. Nevertheless retrieved phase images of the wasp are still shown with a drastic shortening of the acquisition time (a factor 200 between the wasp image done at 2.7 × 10^17^ W/cm^2^ and the one done at 1.3 10^19^ W/cm^2^) thanks to the very high number of K_α_ photons delivered by laser-produced plasma at such high laser driving intensity. Thus, the present study shows a compromise between achieving good image quality and short exposure time depending on the targeted application. This compromise can be balanced by adjusting only one parameter, the laser intensity on target. At low laser intensity, the best x-ray source spatial coherence and the lowest L_s_/${{\rm{L}}}_{\perp }$ ratios are achieved which offer the highest edge contrast signal enhancement, suitable for applications such as the control of material fabrication processes^22^. On the opposite, at very high laser intensity, faster imaging acquisition can be done thanks to high available x-ray photon flux with a L_s_/${{\rm{L}}}_{\perp }$ ratio still below unity.

## Methods

### Data extraction and treatment

**Figure 7:**
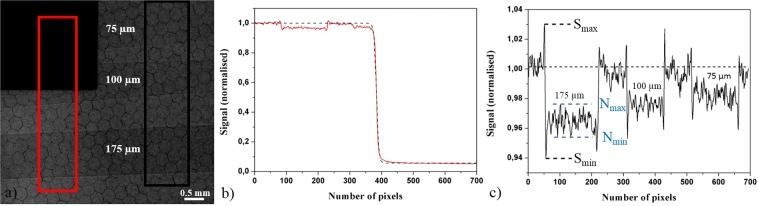
(**a**) Raw image of tungsten knife (black shadow in the left top) and of the sample. Image is acquired at I = 1.0 × 10^18^ W/cm^2^, with 6000 laser shots; the corresponding x-ray source size is 18 µm and the K_α_ x-ray flux 3 × 10^9^ ph/sr/s. The sample is made of PET films of different thicknesses. (**b**) Line intensity profile corresponding to the vertically integrated red area of the ESF. (**c**) Vertically integrated intensity line profile of PET films. Note that this line profile only serves as an example to show the three PET films with different thicknesses in a same graph. Different levels of absorption are visible as well as edge enhanced contrast corresponding to the signature of the refractive index variation between PET and air.

The following procedure to collect the measurements and evaluate the C_edge_ parameter is done for each laser intensity. The K_α_ x-ray flux (Fig. 1b) and the x-ray spectrum (Fig. 1c) are acquired^29^ with a 16 bits direct detection PIXIS-XB: 1024BR camera from Princeton Instruments, cooled down to −60 °C, with 1024 × 1024 pixels arrays and a pixel size of 13 × 13 µm^2^. The sample is then inserted. For imaging applications requiring detector with a large surface, a more suitable detector is used. It is a 16 bits indirect detection CCD camera Quad-RO: 4320 from Princeton Instruments cooled down to −25 °C with 2084 × 2084 pixels and a pixel size of 24 × 24 µm^2^. A raw image of the PET sample and the knife edge is presented in Fig. 7a. In a single image the characterization of the x-ray source size (Fig. 7b) and the extraction of C_edge_ for the three thicknesses of PET films (Fig. 7c) are done. The x-ray source size is extracted thanks to the well-known knife edge technique, using a sharp tungsten knife^29^. The corresponding edge spread function (ESF) is plotted from the acquired image (red line profile of the signal in Fig. 7b). We further determine the x-ray source size by applying a Fermi function fit (black dashed line in Fig. 7b) on the resulted ESF. Moreover, the signal for each PET thickness is extracted in order to calculate a value of C_edge_. The limit of a detectable phase contrast signal is calculated for each image and is equal to C_noise_ = $\frac{{{\rm{N}}}_{{\rm{\max }}}-{{\rm{N}}}_{{\rm{\min }}}}{{{\rm{N}}}_{{\rm{\max }}}+{{\rm{N}}}_{{\rm{\min }}}}\times 100 \%$ (see Fig. 7c). The same procedure is applied for the wasp images.

## Data Availability

The datasets presented in this current study are available from the corresponding author on reasonable request.

## References

[1] Davis TJ, Gao D, Gureyev TE, Stevenson AW, Wilkins SW (1995). Phase-contrast imaging of weakly absorbing materials using hard X-rays. Nature.

[2] Shahani AJ, Xiao X, Voorhees PW (2016). The mechanism of eutectic growth in highly anisotropic materials. Nat. Commun..

[3] Garcea SC, Wang Y, Withers PJ (2018). X-ray computed tomography of polymer composites. Compos. Sci. Technol..

[4] Mayo SC, Stevenson AW, Wilkins SW (2012). In-Line Phase-Contrast X-ray Imaging and Tomography for Materials Science. Materials.

[5] Arfelli F (1998). Low-dose phase contrast x-ray medical imaging. Phys. Med. Biol..

[6] Arfelli F (2000). Mammography with synchrotron radiation: phase-detection techniques. Radiology.

[7] Olivo A (2009). Phase contrast imaging of breast tumours with synchrotron radiation. Appl. Radiat. Isot..

[8] Wilkins SW, Gureyev TE, Gao D, Pogany A, Stevenson AW (1996). Phase-contrast imaging using polychromatic hard X-rays. Nature.

[9] Shovkun VY, Kumakhov MA (2006). Phase contrast imaging with micro focus X-ray tube. Proc. SPIE.

[10] Chen L, Zheng L, Ai-Min Y, Cheng-Quan L (2007). Influence of tube voltage and current on in-line phase contrast imaging using a microfocus x-ray source. Chinese Phys..

[11] Kotre CJ, Robson KJ (2014). Phase-contrast and magnification radiography at diagnostic X-ray energies using a pseudo-microfocus X-ray source. Br. J. Radiol..

[12] Espes E (2014). Liquid-metal-jet x-ray tube technology and tomography applications. Proc. SPIE.

[13] Snigirev A, Snigireva I, Kohn V, Kuznetsov S, Schelokov I (1995). On the possibilities of x‐ray phase contrast microimaging by coherent high‐energy synchrotron radiation. Rev. Sci. Instrum..

[14] Cloetens P, Barrett R, Baruchel J, Guigay J-P, Schlenker M (1996). Phase objects in synchrotron radiation hard x-ray imaging. J. Phys. D: Appl. Phys..

[15] Cloetens P (1999). Hard x-ray phase imaging using simple propagation of a coherent synchrotron radiation beam. J. Phys. D: Appl. Phys..

[16] Spanne P, Raven C, Snigireva I, Snigirev A (1999). In-line holography and phase-contrast microtomography with high energy x-rays. Phys. Med. Biol..

[17] Kneip S (2011). X-ray phase contrast imaging of biological specimens with femtosecond pulses of betatron radiation from a compact laser plasma wakefield accelerator. Appl. Phys. Lett..

[18] Fourmaux S (2012). Laser wakefield acceleration: application to Betatron x-ray radiation production and x-ray imaging. Proc. SPIE.

[19] Najmudin Z (2014). Compact laser accelerators for X-ray phase-contrast imaging. Phil. Trans. R. Soc. A.

[20] Wenz J (2015). Quantitative X-ray phase-contrast microtomography from a compact laser-driven betatron source. Nat. Commun..

[21] Chaulagain U (2017). X-ray phase contrast imaging of biological samples using a betatron x-ray source generated in a laser wakefield accelerator. Proc. SPIE.

[22] Hussein AE (2019). Laser-wakefield accelerators for high-resolution X-ray imaging of complex microstructures. Sci. Rep..

[23] Guo B (2019). High-resolution phase-contrast imaging of biological specimens using a stable betatron X-ray source in the multiple-exposure mode. Sci. Rep..

[24] Ikeura-Sekiguchi H (2008). In-line phase-contrast imaging of a biological specimen using a compact laser-Compton scattering-based x-ray source. Appl. Phys. Lett..

[25] Oliva P (2010). Quantitative evaluation of single-shot inline phase contrast imaging using an inverse compton x-ray source. Appl. Phys. Lett..

[26] Gradl R (2017). Propagation-based Phase-Contrast X-ray Imaging at a Compact Light Source. Sci. Rep..

[27] Gradl R (2018). *In vivo* Dynamic Phase-Contrast X-ray Imaging using a Compact Light Source. Sci. Rep..

[28] Fourmaux S, Kieffer JC (2016). Laser-based Kα X-ray emission characterization using a high contrast ratio and high-power laser system. Appl. Phys. B.

[29] Azamoum Y (2018). High photon flux Kα Mo x-ray source driven by a multi-terawatt femtosecond laser at 100 Hz. Opt. Lett..

[30] Huang K (2014). Intense high repetition rate Mo Kα x-ray source generated from laser solid interaction for imaging application. Rev. Sci. Instrum..

[31] Toth R (2007). Evaluation of ultrafast laser-based hard x-ray sources for phase-contrast imaging. Phys. Plasmas.

[32] Rousse A (2001). Non-thermal melting in semiconductors measured at femtosecond resolution. Nature.

[33] Krol A (2007). Initial experimentation with in-line holography x-ray phase-contrast imaging with an ultrafast laser-based x-ray source. Proc. SPIE.

[34] Martín L (2019). Commissioning of a laser-plasma x-ray micro-focus source for phase contrast imaging. Proc. oSPIE.

[35] Toth R, Kieffer JC, Fourmaux S, Ozaki T, Krol A (2005). In-line phase-contrast imaging with a laser-based hard x-ray source. Rev. Sci. Instrum..

[36] Chakera JA, Ali A, Tsui YY, Fedosejevs R (2008). A continuous kilohertz Cu Kα source produced by submillijoule femtosecond laser pulses for phase contrast imaging. Appl. Phys. Lett..

[37] Laperle CM (2008). Low density contrast agents for x-ray phase contrast imaging: the use of ambient air for x-ray angiography of excised murine liver tissue. Phys. Med. Biol..

[38] Chen LM (2007). Phase-contrast x-ray imaging with intense ArKα radiation from femtosecond-laser-driven gas target. Appl. Phys. Lett..

[39] Li M (2017). Laser-driven powerful kHz hard x-ray source. Radiat. Phys. Chem..

[40] Barbato F (2019). Quantitative phase contrast imaging of a shock-wave with a laser-plasma based X-ray source. Sci Rep.

[41] Pikuz TA (2009). Propagation-based phase-contrast enhancement of nanostructure images using a debris-free femtosecond-laser-driven cluster-based plasma soft x-ray source and an LiF crystal detector. Appl. Opt., AO.

[42] Clady R (2018). 22 W average power multiterawatt femtosecond laser chain enabling 10^19^ W/cm^2^ at 100 Hz. Appl. Phys. B.

[43] Azamoum Y (2018). Impact of the pulse contrast ratio on molybdenum Kα generation by ultrahigh intensity femtosecond laser solid interaction. Sci. Rep..

[44] Fourmaux S (2011). Pedestal cleaning for high laser pulse contrast ratio with a 100 TW class laser system. Opt. Express.

[45] Bravin A, Coan P, Suortti P (2013). X-ray phase-contrast imaging: from pre-clinical applications towards clinics. Phys. Med. Biol..

[46] Momose A (2005). Recent Advances in X-ray Phase Imaging. Jpn. J. Appl. Phys..

[47] Wu X, Liu H (2007). Clarification of aspects in in-line phase-sensitive x-ray imaging. Med. Phys..

[48] Henke BL, Gullikson EM, Davis JC (1993). X-Ray Interactions: Photoabsorption, Scattering, Transmission, and Reflection at E = 50-30,000 eV, Z = 1-92. At. Data Nucl. Data Tables.

[49] Eder DC, Pretzler G, Fill E, Eidmann K, Saemann A (2000). Spatial characteristics of Kα radiation from weakly relativistic laser plasmas. Appl. Phys. B.

[50] Paganin D, Mayo SC, Gureyev TE, Miller PR, Wilkins SW (2002). Simultaneous phase and amplitude extraction from a single defocused image of a homogeneous object. J. Microsc..

[51] Beltran MA, Paganin DM, Pelliccia D (2018). Phase-and-amplitude recovery from a single phase-contrast image using partially spatially coherent x-ray radiation. J. Opt..

[52] Vedantham S, Karellas A (2013). X-ray phase contrast imaging of the breast: Analysis of tissue simulating materials. Med. Phys..

